# Nuclear Eccentricity as a Potential Biomarker for Tumor Biology and Survival in Glioblastoma

**DOI:** 10.3390/curroncol33100627

**Published:** 2026-10-17

**Authors:** Luna Han, Teresia John, Mohid Hassan, Jonathan J. Wisco

**Affiliations:** Department of Anatomy and Neurobiology, Boston University Aram V. Chobanian & Edward Avedisian School of Medicine, Boston, MA 02118, USA; tmjohn@bu.edu (T.J.); mohid@bu.edu (M.H.)

**Keywords:** glioblastoma, nuclear eccentricity, prognosis, biomarker, tumor microenvironment

## Abstract

**Purpose:** To investigate whether quantitative nuclear morphology from routine H&E images can be used as a biomarker for glioblastoma (GBM) biology and prognosis. **Methods:** Nuclear eccentricity, circularity, density, and heterogeneity were measured from H&E whole-slide images in the TCGA-GBM cohort and independently validated in the CPTAC-GBM cohort. A multivariable Cox model was used to test the survival associations in both cohorts, and transcriptome-wide and gene set enrichment analyses were conducted in TCGA RNA data and validated using CPTAC RNA and proteomic data. **Results:** In TCGA-GBM, greater nuclear eccentricity was associated with better survival (HR, 0.85; 95% CI, 0.71–1.02; *p* = 0.089), with the same favorable association independently observed in CPTAC-GBM (HR, 0.74; 95% CI, 0.60–0.90; *p* = 0.003). No individual gene association with eccentricity was statistically significant in the TCGA cohort, but pathway analysis identified its association with epithelial–mesenchymal transition (EMT), inflammatory, hypoxic, and stress-related programs. Across the TCGA RNA, CPTAC RNA and proteomic datasets, greater eccentricity was consistently associated with increased NF-κB signaling, collagen biosynthesis, and ER-to-Golgi transport and decreased L1CAM-related and neuronal-projection programs, with EMT showing the strongest cross-cohort consistency. **Conclusions:** Nuclear eccentricity from patient H&E slide images is a potential biomarker associated with survival and transcriptional/translational programs in GBM. Integrating quantitative pathology with RNA and proteomic data may provide an accessible approach for GBM prognosis and tumor biology characterization.

## Simple Summary

Glioblastoma is an aggressive brain cancer with differences in tumor biology among patients. Although routine pathology slides are available for most patients, biological information contained in specific cell morphology remains understudied. In this study, we measured several features of tumor cell nuclei from standard pathology slides and examined whether they are associated with patient survival and tumor biology in two independent cohorts. We found that greater nuclear eccentricity was associated with better patient survival. It was also consistently associated with several biological processes, including pathways related to tissue remodeling and inflammation, in both RNA and protein datasets. Our findings suggest that information in pathology slides may potentially serve as easily accessible biomarkers for prognosis and biological processes in glioblastoma.

## 1. Introduction

As an aggressive primary brain tumor, glioblastoma (GBM) is generally associated with poor clinical outcomes even after surgical and chemotherapy/radiation treatments [1,2]. GBM is highly heterogeneous among patients, and there are differences in genetic mutation and gene expression patterns between patients and even within individual tumors [3,4,5,6]. Previous studies have found that malignant GBM cells can have four different cellular states—oligodendrocyte progenitor cells (OPC), neural progenitor cells (NPC), astrocytic cells (AC), and mesenchymal cells (MES) [5]. This biological diversity may contribute to treatment resistance; therefore, it is important to identify biomarkers for this tumor heterogeneity.

Histopathology is very important for the diagnosis of GBM because tumors show common morphology such as high cellularity, nuclear pleomorphism, necrosis, and abnormal vascular proliferation [7]. Traditionally, these features are measured visually by pathologists and can be quantified from routine hematoxylin and eosin (H&E)-stained slides with the help of digital pathology [8]. Previous studies have shown that histologic features can be quantified and used to identify different molecular characteristics and clinical outcomes in GBM, and results suggest that tumor morphology may reflect underlying biological states [9,10].

Changes in nuclear size, shape, and density are common in cancer cells; therefore, nuclear morphology may contain particularly useful information, and it may be associated with cell growth, chromatin structure, and even interactions with the surrounding tumor environment [11]. It is also shown that the cellular shape, migration, and proliferation of glioma cells can be affected by the extracellular environment [12]. Because the nucleus responds to these cellular signals, quantification of nuclear shape may provide a useful biomarker for the tumor biology in GBM.

Several previous studies showed that there is a relationship between nuclear morphology and prognosis in GBM, but the direction of this association has not been consistent. Nafe and colleagues reported that nuclear shape was independently associated with survival. More regular and circular nuclei were more common in patients with shorter survival, and irregular nuclear morphology was associated with longer survival in glioblastoma [13,14]. However, Kong and colleagues’ later computational analysis showed a positive association between nuclear circularity and survival. These differences suggest that nuclear shape is useful clinically, but its prognostic interpretation may depend on the context and the specific morphologic measurement used.

Nuclear shape is also linked to the GBM molecular phenotype. It was previously found that higher nuclear circularity and lower eccentricity were associated with oligodendrocyte-related gene expression and proneural characteristics [15,16], suggesting that nuclear morphology may be more than a nonspecific marker for malignancy, rather reflecting differences in tumor cellular states. However, these studies often included nuclear measurement as part of a large image-processing technical framework rather than examining individual features in depth [15,16], and the biological pathways and prognostic values associated with specific nuclear morphology features remain poorly understood.

In this study, we investigated whether nuclear morphologic features quantified from routine H&E-stained GBM tissue are associated with underlying molecular biology and survival outcome. By combining digital pathology with transcriptomic, proteomic, and clinical data from independent GBM cohorts, we assessed the relationships between nuclear morphologic features, biological pathways, and survival, and explored whether routine histologic features could provide accessible biomarkers of glioblastoma heterogeneity and prognosis.

## 2. Methods

### 2.1. Data Source, Image Processing and Nuclear Measurement

Digital hematoxylin and eosin (H&E)-stained whole-slide images were obtained from the National Cancer Institute, Genomic Data Commons (GDC) (www.gdc.cancer.gov, accessed on 1 July 2026) for the Cancer Genome Atlas Glioblastoma Multiforme (TCGA-GBM) and from The Cancer Imaging Archive (TCIA) (www.cancerimagingarchive.net, accessed on 1 July 2026) for the Clinical Proteomic Tumor Analysis Consortium Glioblastoma Multiforme (CPTAC-GBM) cohort. A total of 200 subjects were selected for analysis of nuclear measurement based on the availability of slides for the TCGA-GBM cohort and 164 for the CPTAC-GBM cohort. Slides were matched to clinical, RNA, and proteomic data using available case and specimen identifiers. One primary-tumor H&E slide was utilized for each subject for nuclear morphology measurement, and the slides were analyzed using QuPath [17]. Regions of viable tumors were selected by excluding areas with necrosis, hemorrhage, artifact tissue, or non-neoplastic tissue. Five 0.25 mm^2^ non-overlapping regions of interest (ROIs) were used for each slide. Nuclear detection was conducted using QuPath’s watershed-based cell detection algorithm with a pixel size of 0.5 µm, a minimum nuclear area of 15 µm^2^, a maximum nuclear area of 400 µm^2^, and a detection threshold of 0.15. Nuclear measurements were exported from each ROI and utilized to calculate patient-level nuclear morphology features, including nuclear eccentricity (measuring the elongation of the cell nucleus), nuclear circularity (measuring how round the cell nucleus is), nuclear density (nuclei per unit area), and nuclear heterogeneity, defined as the interquartile range (IQR) divided by the median nuclear area, calculated as (Q3 − Q1)/median. The same image-processing and nuclear measurement method was used for both the TCGA and CPTAC cohorts.

### 2.2. RNA and Proteomic Data Processing

RNA and clinical data for the TCGA-GBM and CPTAC-GBM cohorts were obtained from the GDC. RNA sequencing data were generated using the STAR-counts workflow and were matched to each subject using GDC case and sample identifiers. CPTAC-GBM proteomic data, generated using tandem mass tag (TMT)-based mass spectrometry, were from the Proteomic Data Commons (PDC) and matched to each subject.

Clinical and demographic variables were obtained from GDC and cohort-specific clinical annotation files. Tumor purity estimates were obtained from available ABSOLUTE/GDC-associated purity data and matched to each subject by case identifiers [18]. Imaging, RNA, proteomic, clinical, molecular-marker, and tumor-purity data were then merged at the patient level for analysis.

### 2.3. Survival and Bootstrap Stability Analyses

Cox proportional-hazards regression was used to evaluate the overall survival and progression-free survival after adjusting for age, sex, tumor purity, IDH status, and MGMT promoter methylation status. A total of 179 subjects in the TCGA cohort and 157 subjects for the CPTAC cohort with all covariables available were included in the survival analysis. Incremental prognostic value was assessed by comparing morphology-containing models to the model with only clinical variables. The proportional-hazards assumption was evaluated using Schoenfeld residuals. Model stability was evaluated using a 1000-resample patient-level nonparametric bootstrap. Subjects were sampled with replacement, and the clinical baseline model and morphology models were refitted in each resample using the same covariates. Bootstrap results were presented using median hazard ratios (HRs), 95% percentile intervals, and consistency in the direction of association across resamples.

### 2.4. Transcriptome-Wide Association Using Limma-Voom Framework

The limma-voom framework in R (version 4.6.1, R Foundation for Statistical Computing, Vienna, Austria) was used to test the transcriptome-wide associations between nuclear morphology and gene expression [19]. Lowly expressed genes were excluded, and RNA-seq count data were normalized and transformed using voom. Models were adjusted for variables including age, sex, race, and tumor purity. For each gene, the regression coefficient (β) and *p* value for the morphology variable were estimated using empirical Bayes moderation, and multiple testing was controlled using the Benjamini–Hochberg false discovery rate (FDR).

### 2.5. Gene Set Enrichment and Pathway Analysis

Pathway analysis was conducted using pre-ranked gene set enrichment analysis (GSEA) based on the limma-voom results [20]. Genes were ranked by the moderated t-statistic for each nuclear morphology feature. Positive values indicated higher expression with increasing morphology measurements, and negative values indicated inverse associations. Ranked gene lists were tested against Hallmark, Reactome, and Gene Ontology Biological Process (GO-BP) gene sets from the Molecular Signatures Database (MSigDB) [21]. The normalized enrichment score (NES) was used to summarize the enrichment, with FDR < 0.05 considered significant. Representative significant pathways were displayed according to NES, and selected pathways were further visualized using GSEA running-enrichment plots to show the distribution of pathway genes across the ranked transcriptome.

### 2.6. Statistical Software and Data Analysis

Python (version 3.14.6, Python Software Foundation, Wilmington, DE, USA) and R (version 4.6.1, R Foundation for Statistical Computing, Vienna, Austria) were used for data processing and statistical analyses. Specifically, Python was used for data cleaning; subject-level matching and integration of imaging, molecular, and clinical datasets; calculation and summarization of morphology features; bootstrap analyses; and generating figures. R was used for RNA-sequencing analyses with limma-voom, gene set enrichment and pathway analyses, and Cox proportional-hazards survival modeling.

## 3. Results

### 3.1. Quantitative Nuclear Morphology in TCGA-GBM

Quantitative histopathologic analysis was conducted based on the 200 glioblastoma tumor slides from the TCGA-GBM cohort. The four prespecified nuclear morphology features, nuclear eccentricity, circularity, density and heterogeneity, showed different distributions (Table 1 and Supplementary Figure S1). The mean nuclear eccentricity was 0.80 ± 0.02, and the mean nuclear circularity was 0.73 ± 0.04. Nuclear density showed greater inter-sample variability, with the mean of 3325 ± 1563 nuclei/mm^2^. Nuclear area heterogeneity, defined as the IQR-to-median ratio, had a mean of 0.61 ± 0.09.

As an internal validation of the quantitative morphology measurements, TOP2A and MKI67, two known markers of cellular proliferation [22,23], were included as positive controls for nuclear density. Tumors with higher expression of either gene showed significantly greater nuclear density than those with lower expression. For TOP2A, mean nuclear density was 3756 in the high-expression group vs. 2808 nuclei/mm^2^ in the low-expression group (*p* = 1.0 × 10^−5^). For MKI67, the nuclear densities were 3690 vs. 2963 nuclei/mm^2^ (*p* = 0.0009), respectively. These findings provided internal validation that the image-analysis approach captured the expected differences in nuclear morphology, and the quantitative nuclear measurement is reliable to use in this study.

### 3.2. Association of Nuclear Morphology Features with Overall Survival

**Figure 1:**
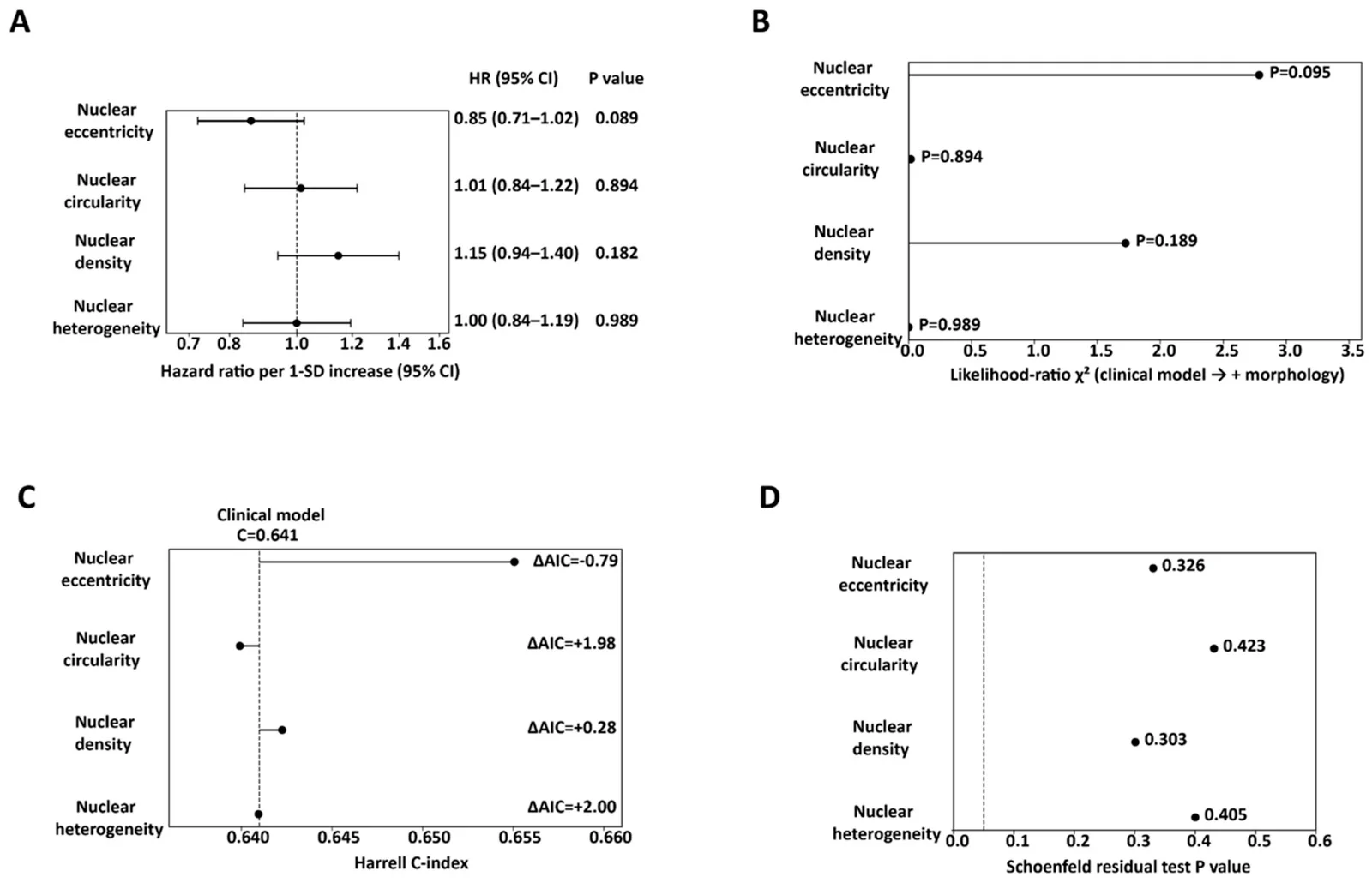
Associations between nuclear morphology features and overall survival. (**A**) Adjusted Cox proportional-hazards models were used to evaluate the association of survival with nuclear eccentricity, circularity, density, and heterogeneity. (**B**) Incremental prognostic contribution of each morphology feature compared to the clinical covariate-only model was assessed using likelihood-ratio χ^2^ statistics. (**C**) Model discrimination and fit assessment using Harrell’s C-index and change in Akaike information criterion (ΔAIC) compared to the clinical model. (**D**) Proportional-hazards diagnostics based on Schoenfeld residual tests; the dashed line indicates *p* = 0.05.

Adjusted Cox proportional-hazards models were used to evaluate the association of the four morphology features with survival in the TCGA-GBM cohort (Figure 1A). Nuclear eccentricity had the strongest association among these four, with greater eccentricity associated with better survival, but the association was not statistically significant (HR, 0.85; 95% CI, 0.71–1.02; *p* = 0.089, N = 179, death = 126). The association between nuclear density and survival was nonsignificant in the opposite direction (HR, 1.15; 95% CI, 0.94–1.40; *p* = 0.182). Nuclear circularity (HR, 1.01; 95% CI, 0.84–1.22; *p* = 0.894) and nuclear heterogeneity (HR, 1.00; 95% CI, 0.84–1.19; *p* = 0.989) also showed no association with survival. The progression-free survival (PFS) analysis showed a directionally similar pattern, and greater nuclear eccentricity was associated with a lower risk of recurrence/progression (HR, 0.87; 95% CI, 0.71–1.07; *p* = 0.188), and nuclear circularity, density, and heterogeneity were not significantly associated with this endpoint (all *p* > 0.4). In the overall survival association model, incremental prognostic contribution showed that the addition of nuclear eccentricity generated the largest improvement over the clinical model (likelihood-ratio χ^2^ approximately 2.8; *p* = 0.095) compared to the other three features (Figure 1B).

Model discrimination was modestly improved by the addition of nuclear eccentricity (Figure 1C). The clinical model had a Harrell’s C-index of 0.641, which increased to 0.655 following inclusion of nuclear eccentricity, with an improvement in model fit (ΔAIC = −0.79). Again, the addition of the other three nuclear features did not improve AIC. Proportional-hazards testing showed no evidence of violation for any of the four morphology variables, with Schoenfeld residual test *p* values of 0.326 for eccentricity, 0.423 for circularity, 0.303 for nuclear density, and 0.405 for nuclear heterogeneity (Figure 1D).

Given that nuclear eccentricity showed the strongest association with overall survival among the four morphology features, its stability was further tested by performing 1000 bootstrap resamples using the same adjusted Cox model. The bootstrap median HR for nuclear eccentricity was 0.84 (95% bootstrap interval, 0.66–1.04), closely matching the observed HR of 0.85. The association remained in the favorable direction (HR < 1) in 94.9% of resamples. These findings indicate that the direction of association between greater nuclear eccentricity and better overall survival was stable across resampling, although the bootstrap interval still spanned the null. Together, nuclear eccentricity showed the most consistent evidence of near-significant association with overall survival among the individual morphology features, supporting its further evaluation in subsequent analyses.

### 3.3. Association of Nuclear Morphology Features with Distinct Transcriptional Profiles

**Figure 2:**
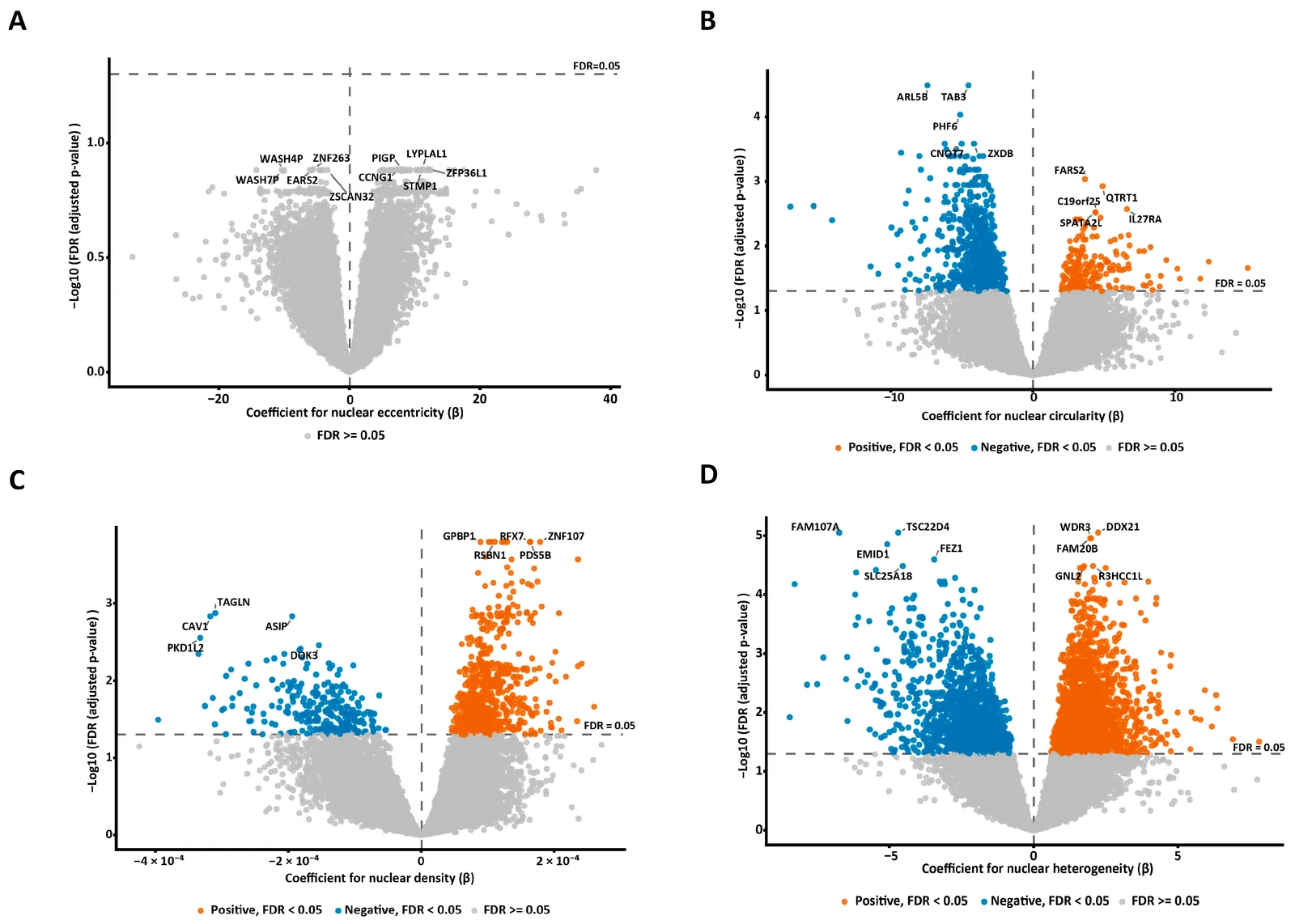
Transcriptome-wide associations between nuclear morphology and gene expression. Volcano plots for the limma-voom regression analysis. (**A**) Nuclear eccentricity, (**B**) nuclear circularity, (**C**) nuclear density, and (**D**) nuclear heterogeneity. The *x*-axis represents the regression coefficient (β) for each morphology feature, and the *y*-axis represents −log10 of the false discovery rate (FDR)-adjusted *p* value. Selected genes with strong associations are labeled. The horizontal dashed line indicates FDR = 0.05.

Transcriptome-wide associations between nuclear morphology and gene expression were evaluated using limma-voom in 192 tumor samples (Figure 2). Nuclear eccentricity showed multiple gene-level associations, but no individual genes reached the significance threshold with FDR < 0.05 (Figure 2A). Nuclear circularity was associated with 921 genes at FDR < 0.05, including 210 positively and 711 negatively associated genes (Figure 2B). Nuclear density was significantly associated with 960 genes, including 692 positive and 268 negative associations (Figure 2C). Nuclear area heterogeneity showed a broad transcriptomic association, with 3319 genes reaching FDR < 0.05, including 1802 positively and 1517 negatively associated genes (Figure 2D). Therefore, the extent and direction of transcriptional association were substantially different among the four morphology features. Nuclear heterogeneity demonstrated the largest number of significant gene-level associations, followed by nuclear density and nuclear circularity, whereas nuclear eccentricity did not identify individual genes meeting the FDR threshold. Nuclear eccentricity’s lack of gene-level associations prompted us to broaden gene-level associations to pathway-level associations, searching for other potential associations with this morphology feature.

### 3.4. Gene-Set Enrichment Analysis (GSEA) on Nuclear Eccentricity

**Figure 3:**
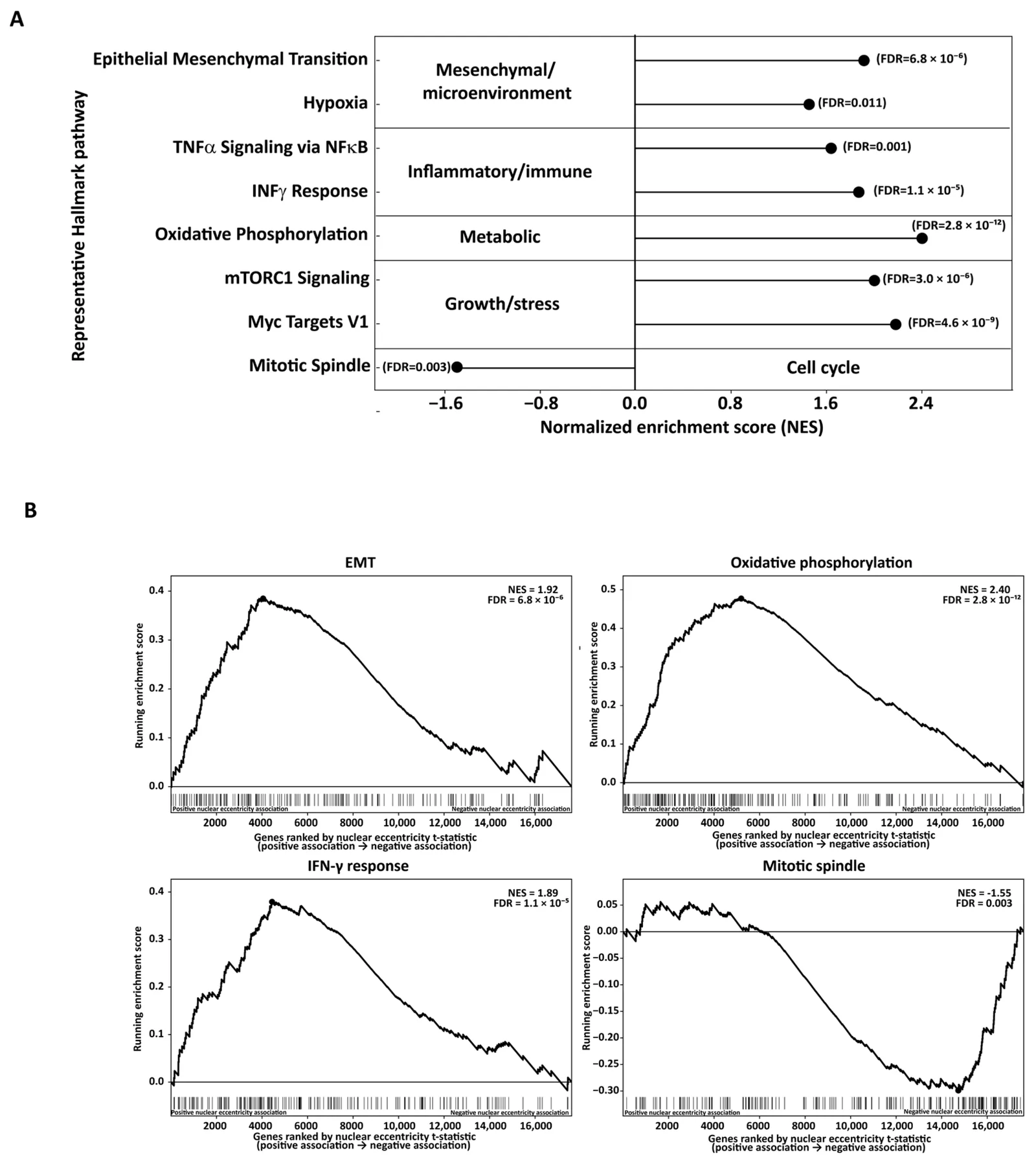
Hallmark pathway enrichment associated with nuclear eccentricity in TCGA. (**A**) Representative Hallmark pathways identified by pre-ranked gene set enrichment analysis (GSEA) using genes ranked by the limma-voom t-statistic for nuclear eccentricity. Pathways are grouped by broad biological functions. Points indicate normalized enrichment scores (NES), with corresponding FDR-adjusted P values. Positive NES indicates enrichment among genes positively associated with greater nuclear eccentricity, and negative NES indicates enrichment among inversely associated genes. (**B**) Representative GSEA enrichment plots for epithelial–mesenchymal transition (EMT), oxidative phosphorylation, interferon-γ response, and mitotic spindle. Vertical tick marks indicate positions of pathway genes within the nuclear eccentricity-ranked transcriptome. NES and FDR values are shown for each pathway.

Coordinated transcriptional changes may exist at the pathway level even when individual genes are not significantly associated. GSEA using the Hallmark gene set was conducted to investigate if any biological programs were associated with nuclear eccentricity (Figure 3A). The pathways positively associated with increased eccentricity included epithelial–mesenchymal transition (EMT; NES = 1.92, FDR = 6.85 × 10^−6^), hypoxia (NES = 1.44, FDR = 0.011), TNFα signaling via NF-κB (NES = 1.65, FDR = 0.001), and interferon-γ response (NES = 1.89, FDR = 1.1 × 10^−5^). Oxidative phosphorylation, mTORC1 signaling, and MYC targets V1 also had positive enrichment, whereas mitotic spindle was negatively enriched (NES = −1.55, FDR = 0.003). Selected GSEA plots indicated that pathways associated with nuclear eccentricity showed coordinated changes across multiple pathway genes rather than isolated strong gene effects (Figure 3B). The distribution of pathway-member genes across the ranked transcriptome supported broad and coordinated pathway-level associations with nuclear eccentricity.

**Figure 4:**
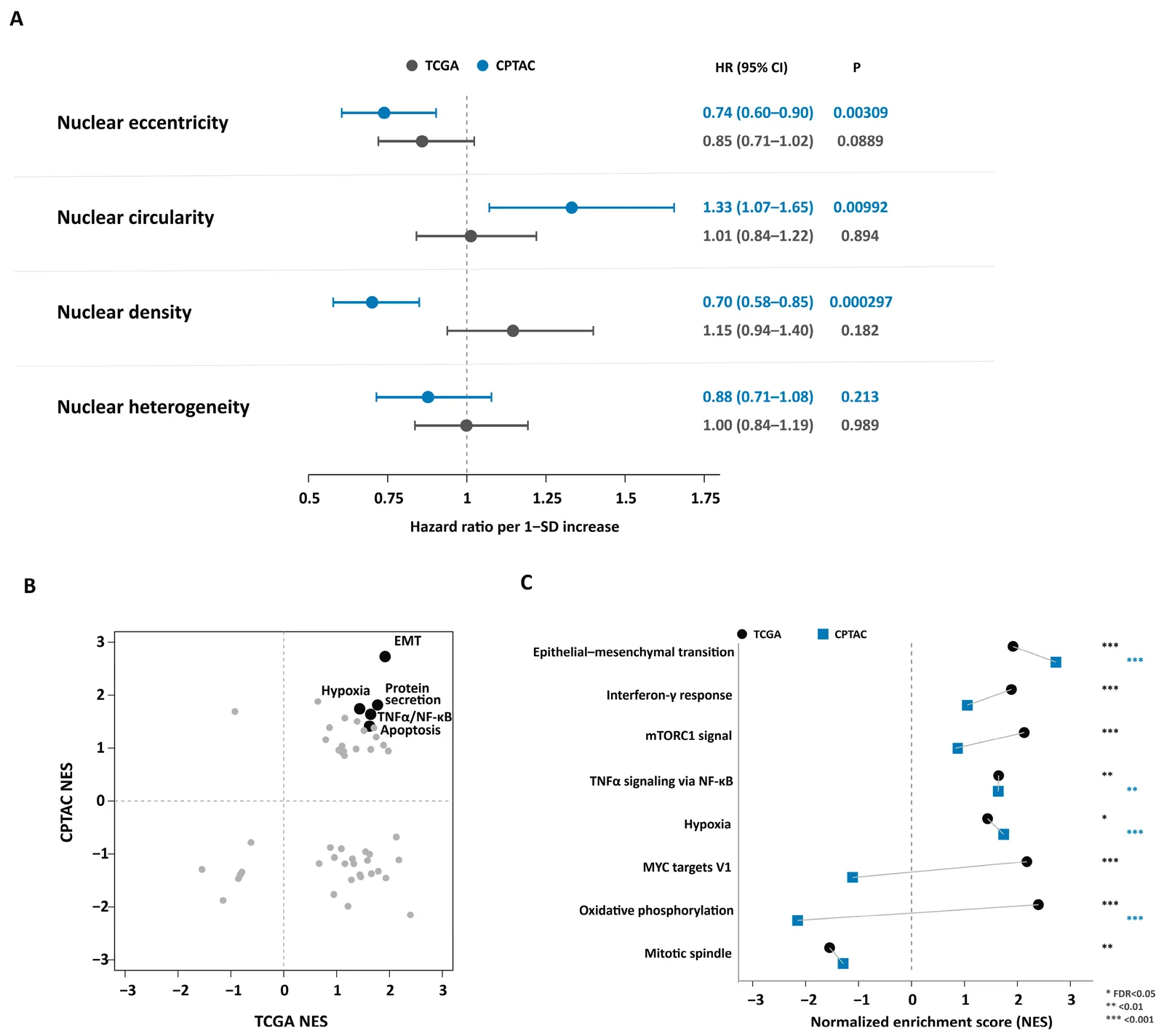
Independent survival and transcriptomic validation of nuclear morphology associations in CPTAC-GBM. (**A**) Adjusted Cox proportional-hazards models comparing associations of nuclear eccentricity, circularity, density, and heterogeneity with overall survival in TCGA and CPTAC. Hazard ratios (HRs) are shown per 1-standard-deviation increase in each morphology feature with 95% confidence intervals. Models were adjusted for age, sex, tumor purity, IDH status, and MGMT status. (**B**) Correlation of nuclear eccentricity-associated Hallmark pathway enrichment across all 50 Hallmark gene sets. Each point represents one pathway, with TCGA NES on the *x*-axis and CPTAC NES on the *y*-axis. EMT, TNFα/NF-κB signaling, hypoxia, protein secretion, and apoptosis are highlighted because they were positively enriched with FDR < 0.05 in both cohorts. Overall Hallmark concordance Spearman ρ = 0.13, *p* = 0.385. (**C**) Comparison of representative Hallmark GSEA normalized enrichment scores for nuclear eccentricity in TCGA and CPTAC RNA. Circles indicate TCGA, and squares indicate CPTAC. Asterisks indicate FDR significance.

The prognostic value of nuclear morphology was further evaluated in the independent CPTAC-GBM cohort (Figure 4). After adjusting for age, sex, tumor purity, IDH status, and MGMT status, the Cox model showed a significant association between greater nuclear eccentricity and better survival (HR = 0.74, 95% CI 0.60–0.90; *p* = 0.003, N = 157, death = 112), and this association was consistent with the TCGA cohort. Nuclear circularity also showed a significant association with survival in CPTAC, with greater circularity associated with increased mortality risk (HR = 1.33, 95% CI 1.07–1.65; *p* = 0.0099), but there was little evidence of an association for this feature in TCGA. Greater nuclear density was associated with improved survival in CPTAC (HR = 0.70, 95% CI 0.58–0.85; *p* < 0.001), and nuclear heterogeneity was not significantly associated with survival. A PFS analysis showed directionally similar associations, with greater nuclear eccentricity having the strongest association with longer PFS (HR, 0.81; 95% CI, 0.67–0.98; *p* = 0.032), but circularity (HR, 1.22; 95% CI, 0.98–1.52; *p* = 0.073), nuclear density (*p* = 0.105), and heterogeneity (*p* = 0.795) showed no significant association. Therefore, nuclear eccentricity showed the most consistent prognostic direction across the two cohorts, while the significant CPTAC associations of nuclear circularity and density appeared more cohort-specific.

CPTAC RNA-GSEA also replicated a subset of the major Hallmark associations found in the TCGA. Five pathways including EMT, TNFα signaling via NF-κB, hypoxia, protein secretion, and apoptosis were positively enriched with FDR < 0.05 in both cohorts (Figure 4B). EMT showed particularly strong cross-cohort consistency, with NES values of 1.92 in TCGA and 2.73 in CPTAC. However, not all pathway associations were the same across the different cohorts (Figure 4C). For example, oxidative phosphorylation showed a strong positive enrichment with greater eccentricity in TCGA (NES = 2.40, FDR = 2.78 × 10^−12^) but a strong negative enrichment in CPTAC RNA (NES = −2.15, FDR = 4.67 × 10^−9^). Consistent with this pathway-specific heterogeneity, correlation across all 50 Hallmark pathways was weak and not statistically significant (Spearman ρ = 0.13, *p* = 0.385). These findings showed selective rather than global transcriptomic replication, with the most consistent cross-cohort associations in mesenchymal, inflammatory, hypoxic, and secretory/stress-related programs.

### 3.5. Proteomics Validation of Nuclear Eccentricity-Associated Biology

**Figure 5:**
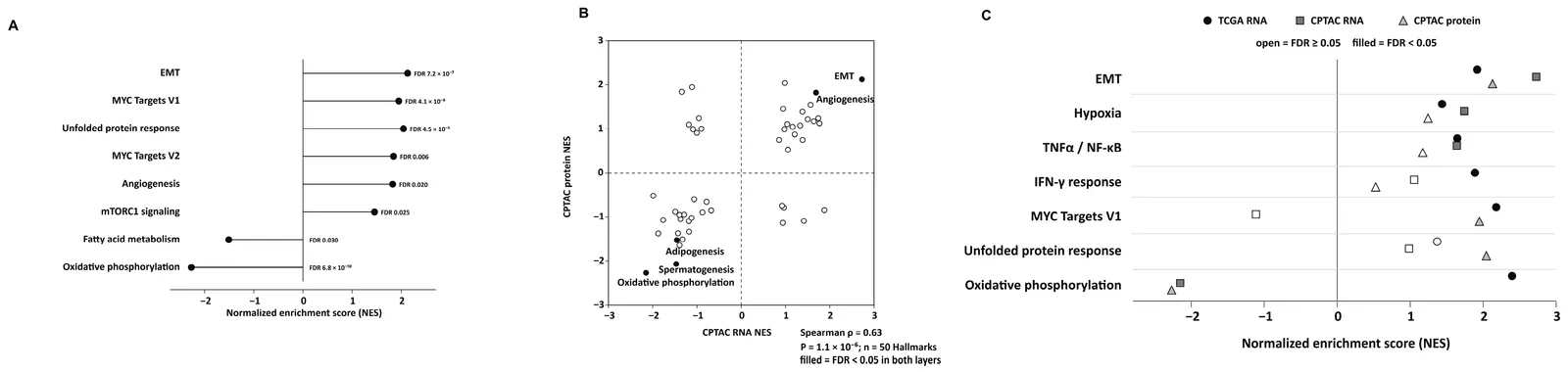
Proteomic validation and RNA–protein correlation of nuclear eccentricity-associated Hallmark pathways in CPTAC-GBM. (**A**) Representative Hallmark pathways associated with nuclear eccentricity in CPTAC proteomic GSEA. Points indicate NES, with corresponding FDR values. (**B**) Global correlation between CPTAC RNA and protein Hallmark enrichment scores for nuclear eccentricity. Each point represents one of 50 Hallmark pathways, with CPTAC RNA NES on the *x*-axis and CPTAC Protein NES on the *y*-axis. Filled points denote pathways meeting FDR < 0.05 in both molecular layers. RNA and protein enrichment scores were positively correlated (Spearman ρ = 0.63, *p* = 1.1 × 10^−6^). Selected pathways are labeled. (**C**) Comparison of selected Hallmark pathway enrichment across TCGA RNA, CPTAC RNA, and CPTAC protein. Black circles indicate TCGA RNA; gray squares indicate CPTAC RNA; and triangles indicate CPTAC protein. Filled symbols indicate FDR < 0.05 and open symbols indicate FDR ≥ 0.05.

Proteome-level GSEA in CPTAC was also conducted to provide an additional molecular layer for evaluating the biology associated with nuclear eccentricity (Figure 5). Greater eccentricity was strongly associated with positive enrichment of EMT (NES = 2.13, FDR = 7.20 × 10^−7^), MYC targets V1, unfolded protein response, MYC targets V2, angiogenesis, and mTORC1 signaling. However, oxidative phosphorylation and fatty-acid metabolism were negatively enriched (Figure 5A). Across the complete set of Hallmark pathways, CPTAC RNA and protein enrichment scores showed a significant correlation (Spearman ρ = 0.63, *p* = 1.1 × 10^−6^) (Figure 5B). Among pathways tested across TCGA RNA, CPTAC RNA, and CPTAC protein, EMT demonstrated the most consistent positive association with nuclear eccentricity (Figure 5C).

### 3.6. Cross-Omic Reactome and GO-BP Validation of Nuclear Eccentricity-Associated Pathways

**Figure 6:**
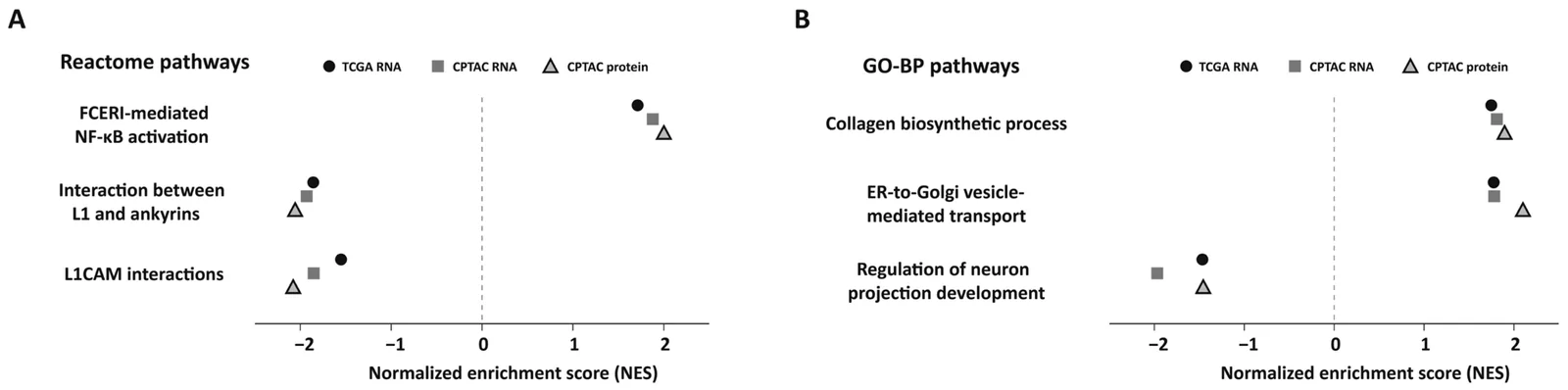
Cross-omic Reactome and Gene Ontology Biological Process validation of nuclear eccentricity-associated biology. Selected pathways showing consistent enrichment direction and FDR < 0.05 across TCGA RNA, CPTAC RNA, and CPTAC protein analyses are shown. (**A**) Reactome pathways. (**B**) Gene Ontology Biological Process (GO-BP) pathways. Black circles indicate TCGA RNA; gray squares indicate CPTAC RNA; and gray triangles indicate CPTAC protein. Positive NES indicates enrichment with greater nuclear eccentricity, and negative NES indicates inverse enrichment.

To investigate more specific biological processes underlying the Hallmark-level findings, Reactome and Gene Ontology Biological Process (GO-BP) datasets were used to compare the TCGA RNA, CPTAC RNA, and CPTAC proteomic datasets (Figure 6 and Table 2). Six pathways showed the same direction of enrichment with FDR < 0.05 in all three datasets. Greater eccentricity was consistently associated with increased FCERI-mediated NF-κB activation, with NES values of +1.71 in TCGA RNA, +1.88 in CPTAC RNA, and +2.00 in CPTAC protein. Two additional positively enriched processes were collagen biosynthetic process and ER-to-Golgi vesicle-mediated transport, supporting coordinated inflammatory, extracellular-matrix, and secretory activity across transcriptomic and proteomic levels.

Conversely, greater eccentricity was consistently associated with reduced neural adhesion and neuronal projection programs. Three negatively enriched pathways include interaction between L1 and ankyrins, L1 cell adhesion molecule (L1CAM) interactions, and regulation of neuron projection development. Together, these cross-cohort and cross-omic findings showed that increasing nuclear eccentricity reflects GBM biology characterized by increased inflammatory/NF-κB, extracellular-matrix, and secretory programs together with reduced neural adhesion and neuronal projection-related programs.

## 4. Discussion

In this study, nuclear morphology features from routine H&E images were integrated with survival, transcriptomic, and proteomic data across two independent GBM cohorts. Nuclear eccentricity, measuring the elongation of the cell nucleus, appears to be the most consistent imaging biomarker. A greater eccentricity was associated with better survival, especially in the CPTAC cohort, and with reproducible biological patterns across the TCGA RNA data and the CPTAC RNA/proteomic data. These findings suggest that a simple feature obtained from routine pathology images may provide valuable information about both prognosis and underlying tumor biology.

The molecular findings were fairly consistent across cohorts and molecular platforms. Although individual gene associations with eccentricity were not statistically significant (Figure 2), pathway analysis revealed broad and biologically coherent patterns. At the Hallmark level, greater eccentricity was associated with mesenchymal/EMT-related, inflammatory, hypoxic, and cellular-stress programs. The Hallmark EMT signature in glioma is generally regarded as reflecting mesenchymal-like biology involving extracellular-matrix remodeling, migration, inflammatory signaling, and cellular plasticity [24,25]. NF-κB signaling is also relevant because activation of this pathway can promote transition toward mesenchymal GBM states and has been linked to invasion and resistance to radiation [26]. Hypoxia can similarly promote mesenchymal transition and invasion through hypoxia-inducible factor-dependent pathways [27]. Therefore, the pathways associated with nuclear eccentricity correspond to several well-established biological processes involved in GBM progression and adaptation.

The Reactome and GO Biological Process analyses further reinforced this pattern. Across the two RNA and one protein datasets, greater nuclear eccentricity was consistently associated with increased pathways such as collagen biosynthesis, inflammatory/NF-κB-related signaling, and ER-to-Golgi transport, and decreased pathways like L1CAM, neuronal projections, and other neural processes. Collagen and extracellular-matrix remodeling are important components of the GBM microenvironment and can support tumor migration and invasion [28,29,30]. Increased ER-to-Golgi transport and unfolded-protein-response signaling may reflect greater secretory and cellular-stress activity, helping GBM cells adapt to hypoxia and metabolic stress [31]. In contrast, reduced neuronal and neuron-projection pathways imply loss of neural or differentiated programs. Together, these findings seem to show a coherent biological theme in which greater eccentricity is associated with stronger mesenchymal, inflammatory, extracellular-matrix, and stress-related programs and weaker neural differentiation programs. The consistency across two independent RNA datasets and an independent proteomic dataset is important because it suggests that the image-derived phenotype reflects biology that is reproducible across different cohorts and molecular layers.

These pathway associations are also relevant to the growing recognition that GBM biomarkers must account for tumor heterogeneity and interactions between malignant cells and the surrounding microenvironment [32]. The previous literature identified four major malignant-cell states in GBM: NPC-like, OPC-like, AC-like, and MES-like. They can coexist within the same tumor and shift in response to genetic and microenvironmental influences [5]. Although our study did not directly assign tumors to the four states, the pathways associated with greater eccentricity, including mesenchymal signaling, inflammatory/NF-κB pathways, hypoxia, extracellular-matrix remodeling, and reduced neuronal programs, are broadly consistent with a shift away from neural or OPC/NPC-like biology and toward MES-like biology. These observations imply that nuclear eccentricity may reflect not only intrinsic tumor-cell properties but also the interaction between tumors and microenvironments. Therefore, a histologic feature on routine H&E may serve as an accessible marker of biological states, which can otherwise only be measured using more expensive molecular approaches.

Our findings regarding survival are also consistent with part of the earlier literature. It was reported previously that nuclear morphology was independently associated with survival and that tumors with more irregularly shaped nuclei tended to occur in patients with longer survival [14]. In a subsequent study, the same group again found that patients with shorter survival had more circular nuclei, whereas longer-surviving patients had nuclei with less regular shapes [13]. Although the Fourier-based shape measurements used in these studies are not identical to eccentricity, both studies are consistent with our finding that greater nuclear eccentricity was associated with longer survival.

In contrast, another study reported a different survival relationship in the computational analysis of TCGA pathology images, showing greater nuclear circularity was associated with more favorable survival [16]. They also linked greater circularity and lower eccentricity with OPC-related gene expression and proneural features. Their molecular findings are generally compatible with our observation that increasing eccentricity was associated with reduced neural programs, but their survival results differ from ours. Differences in image-analysis methods, selected tumor regions, clinical adjustment, and historical diagnostic definitions may contribute to these differences. More broadly, these findings suggest that nuclear morphology contains clinically relevant information, but the prognostic meaning of a specific feature may depend on the biological and spatial context.

The favorable survival association of greater eccentricity is particularly interesting because several molecular programs associated with eccentricity, mesenchymal signaling, hypoxia, inflammation, and extracellular-matrix remodeling are generally considered features of aggressive GBM. Mesenchymal differentiation has been associated with treatment resistance and adverse tumor behavior, and NF-κB-mediated transition toward a mesenchymal state can increase radioresistance [25,26]. This apparent contradiction may reflect the complexity of GBM rather than a true inconsistency. Bulk RNA and protein measurements represent mixtures of malignant cells, immune cells, vascular cells, and extracellular matrix (ECM); therefore, some inflammatory or ECM signals may reflect the composition of the tumor microenvironment rather than intrinsic aggressiveness of the malignant cells [33]. Mesenchymal-like biology is also heterogeneous and can arise through hypoxia, inflammatory signaling, and other microenvironmental influences. Nuclear eccentricity may therefore integrate several biological processes simultaneously, including differentiation, chromatin organization, mechanical interactions with the matrix, and spatial tumor architecture [34]. Its relationship with outcome may not follow the direction predicted by any single molecular pathway. The similar favorable direction of the eccentricity-survival association in the TCGA and CPTAC cohorts supports its potential prognostic relevance, although the mechanism underlying this association remains to be defined.

The definition of GBM has also changed since the TCGA and CPTAC cohorts were assembled. Historically, GBM was mainly defined by histologic features and included both IDH-wildtype and IDH-mutant grade 4 tumors. Under the 2021 WHO classification, the diagnosis of glioblastoma is restricted to IDH-wildtype adult diffuse astrocytic tumors, and tumors previously classified as “IDH-mutant glioblastoma” are now termed astrocytoma, IDH-mutant, CNS WHO grade 4 [1]. IDH-wildtype diffuse astrocytic tumors may also meet criteria for GBM with molecular alterations such as EGFR amplification [1,35]. Because TCGA and CPTAC were established before this classification, their GBM populations do not perfectly match the current disease definition. However, our sensitivity analysis using only IDH-wildtype tumors showed a similar survival pattern, suggesting that the eccentricity findings were not driven by inclusion of IDH-mutant tumors and remain relevant to contemporary GBM. Besides IDH mutation and MGMT methylation status, other molecular markers, such as ATRX loss and 1p/19q codeletion, are also important for distinguishing IDH-mutant astrocytoma from oligodendroglioma [36]. However, these markers were not consistently available in the TCGA and CPTAC cohorts we used, and they were not included in our analysis.

Our findings have potential clinical implications for biomarker development in neuro-oncology. H&E slides are routinely generated for surgically removed samples in GBM and can provide spatial information across a much larger tissue area than most molecular assays. Quantitative morphology therefore offers a potential way to extract additional biomarker information from existing clinical material without requiring additional tissue or specialized testing. This may be particularly useful in a tumor like GBM characterized by great regional heterogeneity since a single molecular sample represents only a limited portion of the tumor. Computational pathology studies have already shown that histologic images contain information related to tumor genotype, transcriptional subtype, and outcome [9,16]. Our results extend this concept by showing that a nuclear feature like eccentricity can be linked not only to prognosis but also to transcriptomic and proteomic programs. Conventional H&E images focus on features such as hypercellularity, necrosis, and pseudopalisading in glioblastoma [37]. Among the measurements we used here, nuclear density mostly reflects cellularity, but nuclear eccentricity and circularity mainly measure the nuclear shape that is not routinely quantified by visual assessment. Therefore, these morphologic features are only prognostic rather than treatment-predictive, and they are best viewed as complementary to the established molecular and pathologic markers, but not as a replacement. If our results are validated in a future prospective study, they may provide a low-cost adjunctive measurement for stratifying biologically different groups.

## 5. Limitations

There are several limitations to this study. First, both TCGA and CPTAC are retrospective cohorts, and differences in tissue processing, treatment, and patient selection may influence the results. We reduced this concern by applying the same image-analysis approach to both cohorts and independently validating the main findings in CPTAC. Second, nuclear measurements were derived from selected viable-tumor regions and therefore cannot capture the full spatial heterogeneity of GBM. To limit sampling bias, up to five non-overlapping viable-tumor ROIs were analyzed per case, although region-level variation may still need further study. A formal interobserver reproducibility analysis of region selection may still be warranted in the future. Third, RNA and proteomic measurements were obtained from bulk tissue, making it difficult to assign pathway signals to specific cell populations. We adjusted for tumor purity and focused on the pathways reproduced across independent RNA datasets and proteomic data, but the relationship between eccentricity and Neftel cell states remains preliminary. Pathway enrichment also reflects association rather than causation, and some pathways such as oxidative phosphorylation were inconsistent between cohorts; therefore, we focused on the most reproducible cross-cohort and cross-omic signals. Finally, historical TCGA and CPTAC diagnoses do not fully match current WHO criteria. However, adjustment for IDH status and sensitivity analyses restricted to IDH-wildtype tumors showed similar overall results. Future studies using prospective multi-institutional IDH-wildtype GBM cohorts will be needed to verify the results.

## 6. Conclusions

In conclusion, nuclear eccentricity measured from routine H&E images was positively associated with survival and with reproducible molecular patterns across independent GBM cohorts and across transcriptomic and proteomic platforms. Higher eccentricity was linked to stronger mesenchymal, inflammatory, extracellular-matrix, stress-related programs and weaker neural differentiation programs. These findings showed that quantitative pathology can provide an interpretable imaging biomarker that connects tumor morphology to tumor biology and clinical outcome. In the future, integrating computational histomorphology with molecular biomarkers may offer a practical approach to capturing heterogeneity and evaluating prognosis in GBM.

## Figures and Tables

**Table 1:** Quantitative nuclear morphology characteristics of the TCGA-GBM cohort.

Nuclear Measurement	Unit	N	Mean	SD	Median	IQR
Nuclear eccentricity	none	200	0.80	0.02	0.80	0.03
Nuclear circularity	none	200	0.73	0.04	0.74	0.05
Nuclear density	nuclei/mm^2^	200	3325	1563	3046	1933
Nuclear area heterogeneity	IQR/median	200	0.61	0.09	0.60	0.11

Baseline summary statistics for nuclear eccentricity, circularity, density, and heterogeneity were calculated for the 200 samples from the TCGA analysis. Results are presented as mean ± standard deviation (SD) and median (interquartile range, IQR).

**Table 2:** Cross-omic Reactome and Gene Ontology Biological Process pathway concordance associated with nuclear eccentricity.

Pathway	Database	TCGA RNA	CPTAC RNA	CPTAC Protein
Inflammatory/ECM/secretory programs ↑				
FCERI-mediated NF-κB activation	Reactome	↑ +1.71 *	↑ +1.88 ***	↑ +2.00 ***
Collagen biosynthetic process	GO-BP	↑ +1.75 *	↑ +1.81 **	↑ +1.90 *
ER-to-Golgi vesicle-mediated transport	GO-BP	↑ +1.77 **	↑ +1.78 **	↑ +2.10 ***
Neural adhesion/projection programs ↓				
Interaction between L1 and ankyrins	Reactome	↓ −1.86 *	↓ −1.93 **	↓ −2.06 ***
L1CAM interactions	Reactome	↓ −1.55 *	↓ −1.85 ***	↓ −2.08 ***
Regulation of neuron projection development	GO-BP	↓ −1.47 *	↓ −1.97 ***	↓ −1.46 *

Normalized enrichment scores for selected Reactome and GO-BP pathways associated with nuclear eccentricity across TCGA RNA, CPTAC RNA, and CPTAC protein analyses. Pathways were included when the direction of enrichment was concordant and FDR < 0.05 in all three datasets. Upward and downward arrows indicate positive and negative enrichment with increasing nuclear eccentricity, respectively. Selection criterion: same NES direction and FDR < 0.05 in all three datasets. * FDR < 0.05; ** FDR < 0.01; *** FDR < 0.001.

## Data Availability

Data are available from the authors upon request.

## Supplementary Materials

The following supporting information can be downloaded at: https://www.mdpi.com/article/10.3390/curroncol33100627/s1, Figure S1: Representative examples of low and high nuclear eccentricity in the TCGA glioblastoma cohort.
