# Supplementary material for: Nuclear Eccentricity as a Potential Biomarker for Tumor Biology and Survival in Glioblastoma
Source: Curr Oncol. 2026 Oct 17;33(10):627. doi: 10.3390/curroncol33100627 (PMC13702909; doi:10.3390/curroncol33100627)
Supplement: Supplementary file 1 [file curroncol-33-00627-s001.zip › curroncol-4564591-supplementary.pdf]

Supplementary Material

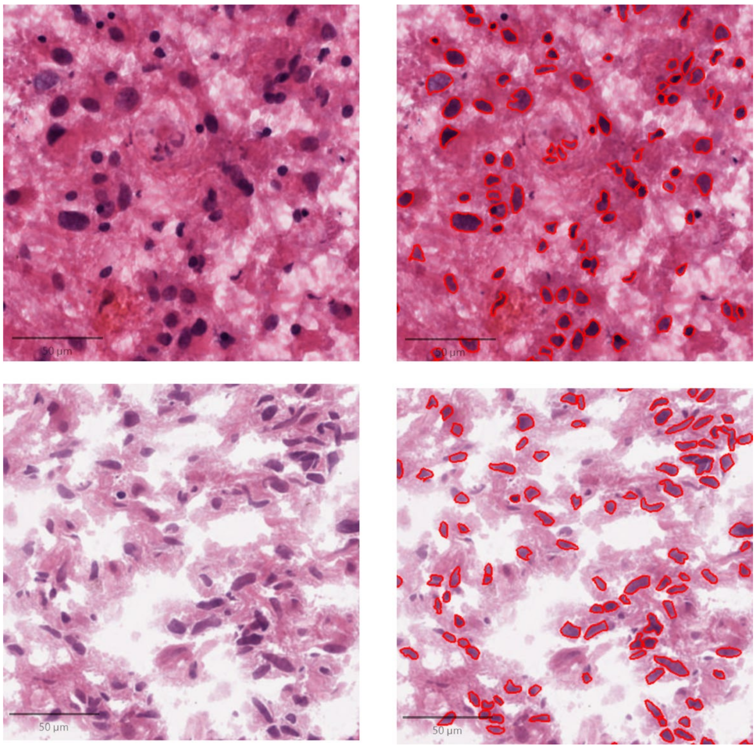

Figure S1. Representative examples of low and high nuclear eccentricity in the TCGA glioblastoma cohort. Representative H&E images showing tumor regions with relatively low (top) and high (bottom) nuclear eccentricity. Red contours on the right indicate nuclei identified by the QuPath cell-detection workflow used for automated morphometric analysis. Scale bars, 50 μm. Nuclear eccentricity measures the elongation of the cell nucleus, and nuclear circularity measures how round the cell nucleus is.
